# Living together: evolutionary and ecological dimensions of protist endosymbiosis

**DOI:** 10.1093/femsml/uqag013

**Published:** 2026-04-06

**Authors:** Anna Karnkowska, Iván García-Cunchillos, Marta Sałek

**Affiliations:** Faculty of Biology, Institute of Evolutionary Biology, University of Warsaw, ul. Żwirki i Wigury 101, 02-089Warsaw, Poland; Faculty of Biology, Institute of Evolutionary Biology, University of Warsaw, ul. Żwirki i Wigury 101, 02-089Warsaw, Poland; Faculty of Biology, Institute of Evolutionary Biology, University of Warsaw, ul. Żwirki i Wigury 101, 02-089Warsaw, Poland; Faculty of Biology, Institute of Evolutionary Biology, University of Warsaw, ul. Żwirki i Wigury 101, 02-089Warsaw, Poland

**Keywords:** anaerobiosis, diazotrophs, microbial eukaryotes, organellogenesis, photosymbiosis, symbiosis

## Abstract

Protists, which comprise the majority of eukaryotic diversity, frequently engage in endosymbiotic relationships with other unicellular eukaryotes or prokaryotes. These interactions have profoundly shaped eukaryotic evolution, not only through the origin of endosymbiotic organelles and the subsequent diversification of eukaryotes, but also via less studied endosymbioses that have influenced the evolution of diverse eukaryotic lineages. Endosymbioses often alter host metabolic capabilities, enabling the colonisation of new ecological niches and significantly contributing to ecosystem functioning. In recent years, interest in these interactions has increased, driven by methodological innovations and new discoveries that reveal the diversity, mechanisms, and ecological roles of protist endosymbioses. Despite these advances, key questions remain: How widespread and ecologically impactful are protist endosymbioses? What functions do symbionts provide, and how do associations form, persist, or break down? Addressing these questions requires systematic studies of protists in their natural environments, combining microscopy and sequencing using both high-throughput and single-cell approaches, along with experimental manipulations of host-symbiont interactions. Here, we review current knowledge, highlight recent breakthroughs, and discuss ongoing challenges in the study of protist endosymbioses.

## Teaser

From symbionts to organelles: evolutionary integration of microbial partners into eukaryotic cells.

## Introduction

**Figure 1:**
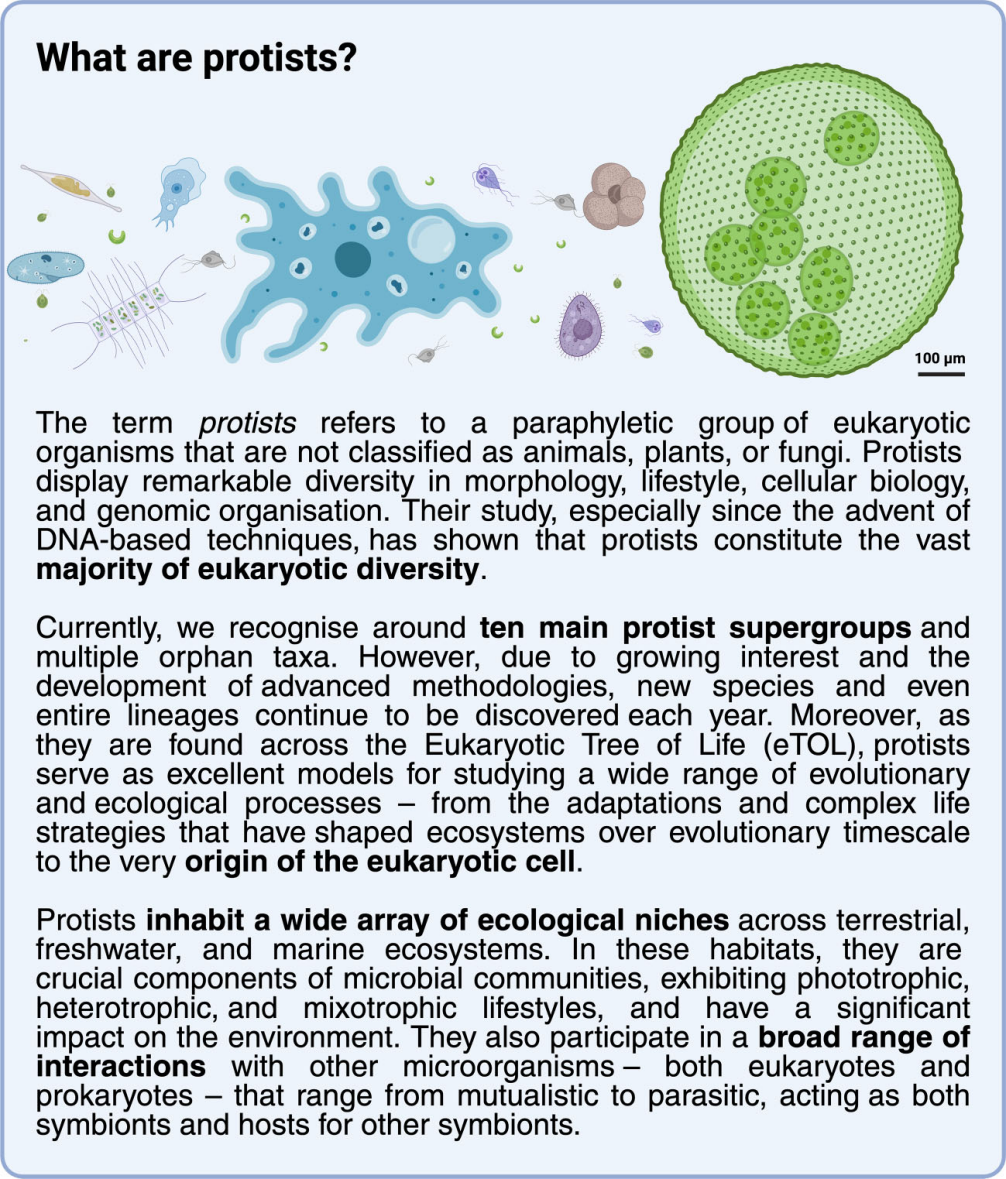
Protists—definition, diversity, and ecological roles. Iconographical representation of size and morphological differences among protists.

Protists (Fig. 1), also known as microbial eukaryotes, are among the least-studied components of microbial communities and research on them continues to lag behind that on bacteria (Keeling and del Campo 2017, Schoenle et al. 2025). The emergence and popularisation of high-throughput DNA sequencing technologies, and particularly the metagenomic revolution, have transformed our understanding of prokaryotic diversity, uncovering the vast range of microbial communities and their biology beyond the low percentage of taxa that can be cultured (Nayfach et al. 2021, Nishimura and Yoshizawa 2022). However, comparable progress for protists is more difficult to achieve and only recently have the measurable advances in eukaryotic metagenomics begun to appear (Carradec et al. 2018, Delmont et al. 2022, Alexander et al. 2023). Furthermore, inferring eukaryotic biology solely from genomic data is usually challenging and advanced microscopy, single-cell approaches, and cultivation are often required to resolve their life cycles, interactions (Alacid and Richards 2021), and cell biology (Dacks and Ginger 2023).

Although some protists have been studied in great detail, these are mainly parasitic species (Lepper et al. 2025), and represent only a small fraction of the true diversity and biological complexity of this group (Burki et al. 2020, Valt et al. 2026). Nonetheless, our understanding of protist diversity has expanded dramatically in recent years, driven by single-cell approaches, various ‘omics’ analyses, cultivation efforts, and advances in microscopy. Beyond well-characterised groups such as diatoms (Stramenopila, TSAR) or kinetoplastids (Discoba), these efforts have uncovered numerous new taxa and previously unrecognised major evolutionary lineages, providing crucial insights into the evolutionary history and diversification of eukaryotes (e.g. Lax et al. 2018, Tikhonenkov et al. 2022, Eglit et al. 2024, Valt et al. 2026).

**Figure 2:**
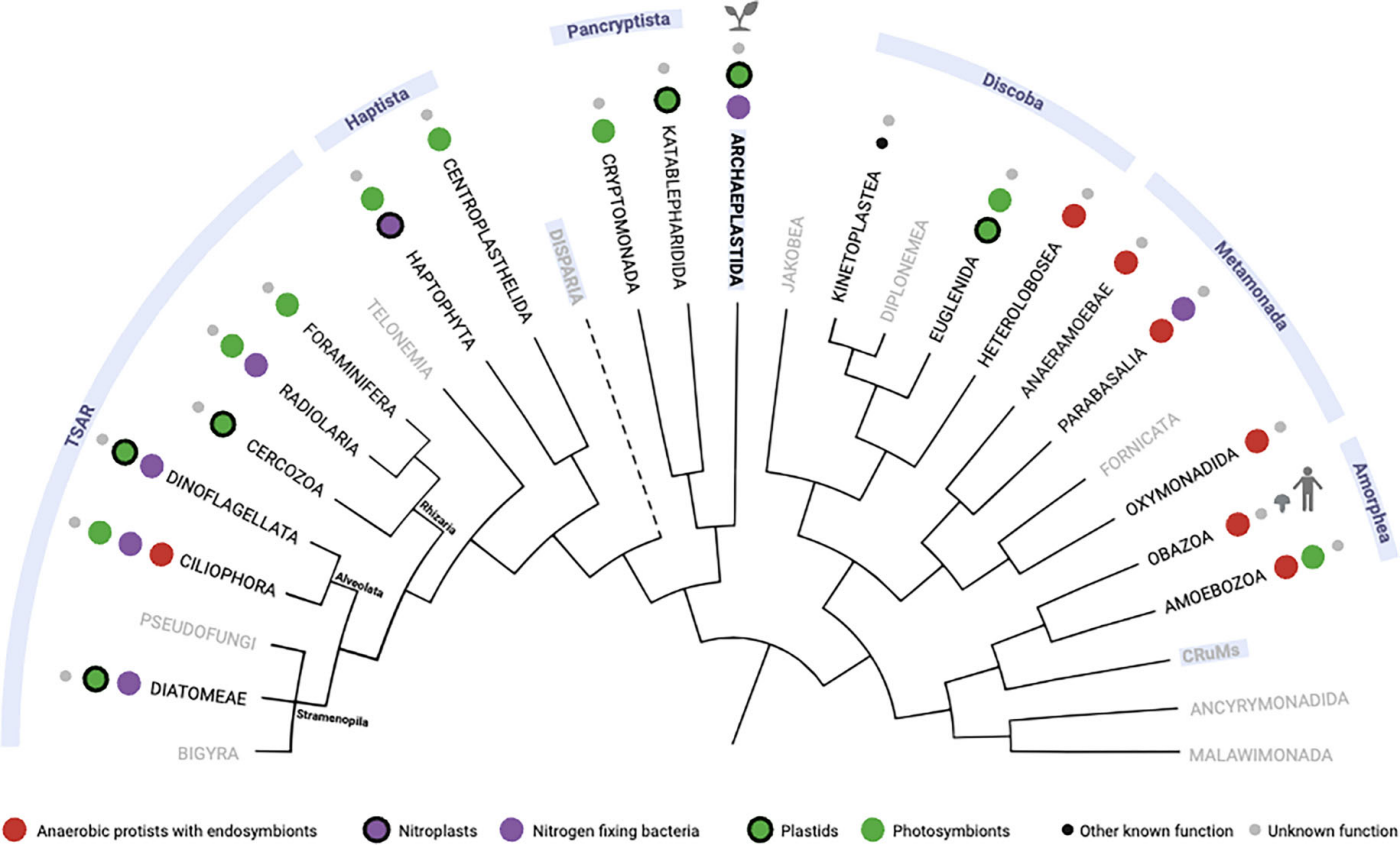
Phylogenetic tree of eukaryotes depicting the diversity of endosymbiotic associations across protist lineages. Major eukaryotic clades (supergroups) are represented according to the current consensus phylogeny, uncertainties are marked with a dashed line (adapted from Burki et al. 2020 and Valt et al. 2026). Organelles and endosymbionts are indicated when at least one example is known for the lineage. Associations in multicellular lineages are omitted. Names of the referenced supergroups are shown against a light-blue background; groups highlighted in black font are those discussed in the paper.

Protists are not solitary members of microbial communities but are constantly interacting with a wide range of other organisms, including other protists, bacteria, and archaea. While some of these interactions are transient, many are long-term symbiotic associations that range from mutually beneficial to parasitic. Among these, intracellular partnerships—endosymbioses—are particularly significant, having played a central role in the evolution and diversification of eukaryotes (Archibald 2015) and influencing ecosystem functioning (e.g. Decelle et al. 2015, Worden et al. 2015, Beinart 2019) (Fig. 2). An additional layer of interaction is provided by protist-infecting viruses, which are increasingly recognised as integral components of microbial ecosystems (Koonin and Yutin 2019, Moniruzzaman et al. 2020, Queiroz et al. 2024). Members of the Nucleocytoviricota, including algal viruses such as the Phycodnaviridae, infect a broad diversity of protists and are widespread in aquatic environments, where they can regulate host populations through infection-driven lysis and thereby contribute to dissolved organic matter release and nutrient cycling (Suttle 2007, Surgenor and McCormic 2026).

In recent years, endosymbioses involving protist partners have attracted increasing interest, revealing associations across nearly all major protist lineages (Husnik et al. 2021). However, significant gaps remain in our understanding. Certain groups, such as amoebozoans (Amorphea) and ciliates (Alveolata, TSAR), appear especially prone to harbouring endosymbionts (Rotterová et al. 2022, Zhang et al. 2024, Schulz et al. 2025), but it remains unclear whether this reflects a genuine biological pattern or differences in the level of study and detectability. In addition, most endosymbioses have been characterised in laboratory cultures, which allow detailed genomic, microscopic, and functional analyses but are inherently limited: they focus on cultivable strains, and since facultative symbionts may be lost during culture maintenance, the true prevalence of symbiont-harbouring protists in natural populations remains poorly understood.

Addressing these gaps has become increasingly feasible thanks to major methodological advances. High-throughput ‘omic’ approaches, including genomics (McKenna et al. 2024), the targeted analyses of specific protist lineages through their semiautomatic detection via fluorescence-activated cell sorting (FACS) (e.g. Wittmers et al. 2025), and single-cell (meta)genomics (e.g. Schulz et al. 2025, Rotterová et al. 2025), which capture the genomes of individual protists together with their endosymbionts, now enable direct access to host–symbiont genomic diversity within complex communities. In parallel, advances in high-resolution imaging, such as focused ion beam scanning electron microscopy (FIB-SEM), which allows nanoscale three-dimensional reconstruction of cellular ultrastructure (e.g. Jerlström-Hultqvist et al. 2024), and nanoscale secondary ion mass spectrometry (NanoSIMS), which maps elemental and isotopic composition at subcellular resolution (e.g. Tschitschko et al. 2024), provide unprecedented insight into the spatial organisation and potential functions of endosymbionts within their protist hosts. Together, these complementary approaches enable more precise characterisation of symbiont diversity, distribution, and ecological roles. Collectively, they are revealing a more nuanced and dynamic picture of protist–prokaryote interactions, demonstrating that endosymbioses are widespread, ecologically significant, and central to understanding both eukaryotic evolution and the structure and functioning of microbial ecosystems.

In this short review, we focus on symbiotic relationships within protist taxa that have played a central role in the evolution and diversification of eukaryotes, providing the eukaryotic cell with new functions. We also discuss the current state of knowledge regarding the roles of these and other protist-based endosymbioses in ecosystem functioning. We provide an overview of these interactions, highlight their potential to address fundamental biological questions, and outline the key methodological and conceptual challenges that remain.

### Endosymbiosis in the evolution of eukaryotic cells: beyond mitochondria and plastids

Although the order and number of events, as well as the quantity and identity of partners involved in the origin of the eukaryotic cell, remain subjects of ongoing debate, the central role of endosymbiosis in this process is beyond doubt (e.g. Raval et al. 2022). The best examples are undoubtedly the acquisition of the energetic organelles, mitochondria and plastids, each involving different prokaryotes as endosymbionts. The origin of mitochondria as an organelle appears to precede the Last Eukaryotic Common Ancestor (LECA) (e.g. Archibald 2015, Vosseberg et al. 2024), while the origin of the photosynthetic organelles—plastids—was the endosymbiotic event that had the most significant impact on the evolution and diversification of eukaryotes.

Although the impact of acquiring endosymbiotic organelles such as plastids and mitochondria is unparalleled in the evolution of the eukaryotic cell, endosymbioses in microscopic eukaryotes are very diverse and broadly distributed across the eukaryotic tree of life (eTOL) (Nowack and Melkonian 2010, Husnik et al. 2021). In recent years, it has become evident that protists across the tree of eukaryotes host endosymbionts, both prokaryotic (bacterial and/or archaeal) and eukaryotic (Fig. 2).

Many groups of bacteria, and to a lesser extent archaea and unicellular eukaryotes, can form endosymbiotic relationships with protists. A broad diversity of bacteria has been identified as protist endosymbionts, including well-known intracellular lineages such as *Rickettsiales, Holosporineae*, and *Chlamydiota*, as well as representatives of numerous other bacterial groups. In contrast, archaeal symbionts of protists are taxonomically limited and associated with anaerobic protists (Husnik et al. 2021). Protists may also harbour eukaryotic endosymbionts, most notably diverse unicellular photosynthetic algae (Decelle et al. 2015), as well as parasitic species from lineages such as Syndiniales (Alveolata) (Decelle et al. 2022) or *Perkinsela* sp. (Kinetoplastea), an obligate intracellular symbiont of certain amoebozoans (Tanifuji et al. 2017).

**Figure 3:**
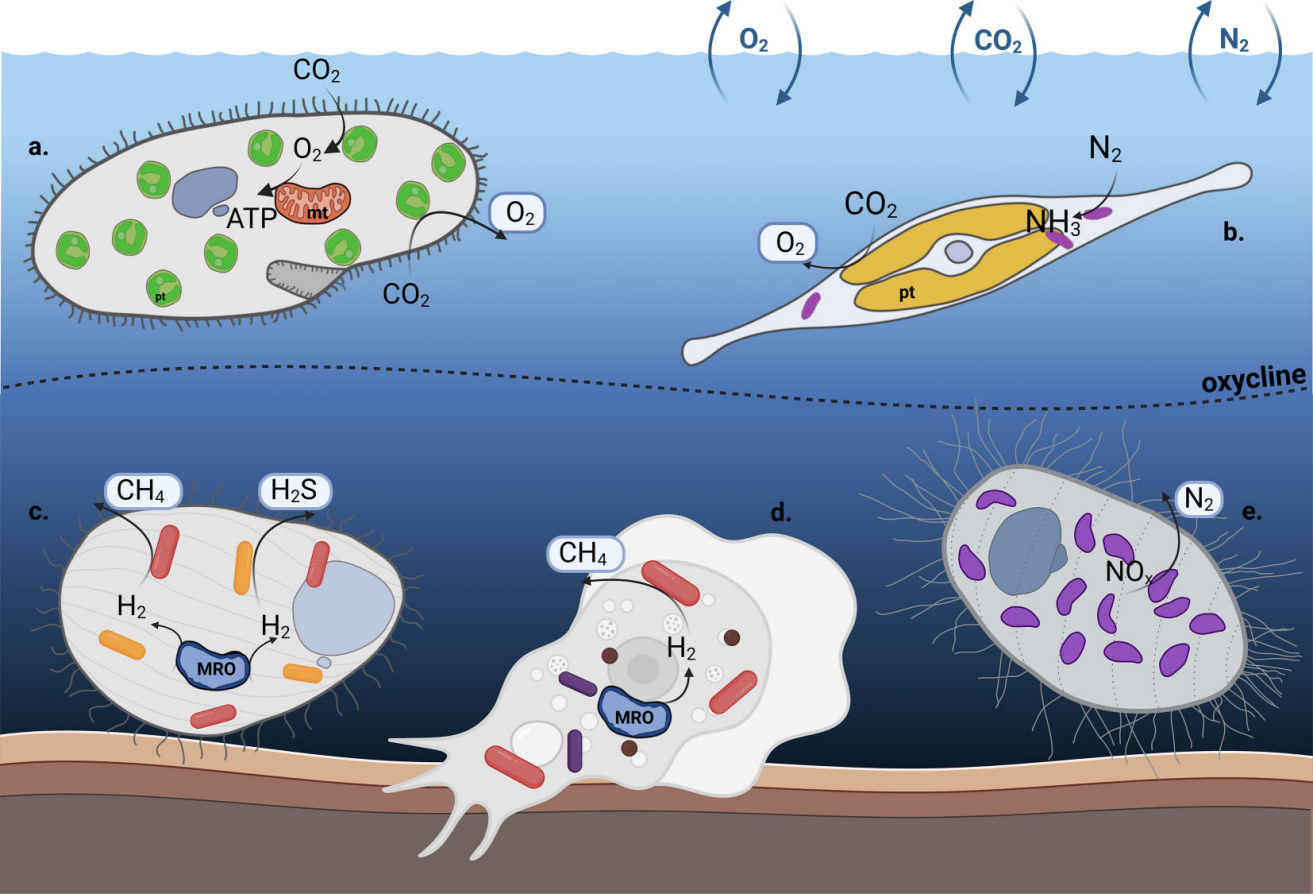
Key functions of protists and their endosymbionts in natural environments. Including photosynthesis (by fully integrated plastids and by algal photosymbionts), nitrogen fixation, denitrification, methanogenesis, and sulphide genesis. mt—mitochondria; pt– plastids; MRO—mitochondria-related organelles) a. *Paramecium bursaria* ciliate with eukaryotic photosymbiont *Chlorella* b. *Epithemia* diatoms with bacterial diazotrophic endosymbionts c. Ciliate with prokaryotic endosymbionts involved in sulphur and methane cycling d. Amoebozoan *Pelomyxa schiedti*, with two bacteria and one methanogenic archaeon as endosymbionts e. Plagiopylean ciliate with endosymbiotic denitrifying bacteria *Ca*. Azoamicus ciliaticola.

As noted above, the prevalence of endosymbiotic interactions across specific protist lineages remains poorly understood and is unknown for many groups. An additional question is whether these interactions are heritable and co-evolve with their hosts, or are short-lived in evolutionary terms. This issue has so far been only partially explored, though it is attracting increasing interest. Studies of ciliates reveal different modes of acquisition of presumably obligate endosymbionts. In the order *Metopida*, e.g. vertical transmission of methanogenic symbionts appears to be the predominant mode, as supported by metagenomic surveys and culture-based experiments, at least over short evolutionary timescales and/or with occasional symbiont switches (Méndez-Sánchez et al. 2024, Rotterova et al. 2025). In contrast, in the ciliate genus *Euplotes*, endosymbionts are acquired primarily through horizontal transmission, with frequent replacements (Boscaro et al. 2019). Together, these results illustrate the remarkable diversity of protist–symbiont interactions, while highlighting several fundamental questions. These include the roles of hosts and symbionts in establishing and maintaining the association, how the underlying mechanisms vary between bacterial, archaeal, and eukaryotic symbionts, how they differ even among lineages within these groups, and the potential role of viruses in shaping these interactions (Song et al. 2024). Metabolic symbioses in protists are among the best characterised, including nutritional interactions, in which symbionts provide essential metabolites to their hosts, and syntrophic associations (i.e. cross-feeding), in which partners rely on each other’s metabolic activities. Nutritional symbioses are particularly well exemplified by photosymbioses, as in the ciliate *Paramecium bursaria* and the green-alga *Chlorella* (Fig. 3a), where the host offers protection and nutrients, while the algal symbiont supplies sugars (Jenkins 2024). Other examples include insect-associated trypanosomatids (e.g. *Angomonas*) (Kinetoplastea), which rely on betaproteobacterial endosymbionts for the supply of purines, haem, amino acids, and vitamins (Harmer et al. 2018). Syntrophic symbioses constitute another well-characterised category. The classic example is the interaction of anaerobic ciliates and their methanogenic archaeal partners, in which metabolic cooperation is essential for energy generation under low-oxygen conditions (Beinart et al. 2018b). Although the number of identified endosymbioses has grown dramatically in the past decade (Fig. 2), the functions of most remain poorly understood. In many cases, symbionts drive the interaction with little or no apparent benefit to the host (so-called “professional symbionts”; e.g. Castelli et al. 2022, Giannotti et al. 2022, Hollender et al. 2024), whereas in other systems, they can modulate host physiology and fitness by altering metabolic processes or providing defence against viruses, with effects that shift depending on environmental conditions or evolutionary context (e.g. Grosser et al. 2018, König et al. 2019, Arthofer et al. 2022). Metabolic symbioses are not necessarily the most common among protist endosymbioses, yet they have profoundly influenced eukaryotic diversification, enabling hosts to exploit new ecological niches.

### Symbiosis and the rise of photosynthetic eukaryotes

The acquisition of plastids represents the most transformative endosymbiosis between eukaryotes and prokaryotes. In this process, a eukaryotic cell acquired a photosynthetic cyanobacterial endosymbiont related to the extant deep-branching species *Gloeomargarita lithophora*, and most likely occurred in terrestrial-freshwater environments (Ponce-Toledo et al. 2017) over 1.5 billion years ago (Yoon et al. 2004). Plastids provided the eukaryotic cell with the capacity for autophototrophy, followed by an explosion in species diversification, currently estimated at between 450 000 and 500 000 species (Bowles et al. 2023) that gave rise to the Archaeplastida clade encompassing land plants and green, red, and glaucophyte algae (Rodríguez-Ezpeleta et al. 2005), as well as some secondarily plastid-lacking taxa (Gawryluk et al. 2019, Schön et al. 2021). However, the impact of this primary endosymbiosis extends beyond Archaeplastida. Subsequently, multiple clades of microscopic eukaryotes independently acquired ‘complex’ plastids (Fig. 2), involving secondary endosymbioses and the subsequent reduction of various red and green algae from endosymbionts to organelles (e.g. McFadden et al. 2001, Takahashi et al. 2007, Keeling 2013, Pietluch et al. 2024). Diversification following these secondary endosymbioses was also noteworthy, leading to the formation of multiple diverse and ecologically relevant clades, including diatoms, dinoflagellates (Alveolata, TSAR), haptophytes (Haptista), cryptomonads (Pancryptista), and euglenids (Discoba).

The evolutionary history of autophototrophy in eukaryotes includes a second, independent acquisition of photosynthetic organelles in some species of the genus *Paulinella*, a clade of microscopic, unicellular cercozoan amoebae (Cercozoa, Rhizaria, TSAR) (Marin et al. 2005, Nowack et al. 2008). Molecular data indicate that, unlike the plastids in Archaeplastida, *Paulinella* acquired their primary plastids (sometimes referred to as chromatophores) independently 90–140 million years ago (Delaye et al. 2016) from a different group of cyanobacteria, related to the clade *Synechococcus*/*Prochlorococcus* (Marin et al. 2005). Despite the recent description of new plastid-bearing *Paulinella* species (Lhee et al. 2019, Gabr et al. 2020), a massive diversification following organelle acquisition does not appear to have occurred so far. The study of *Paulinella* plastids is providing an unprecedented understanding of the process of organelle acquisition (e.g. Nowack et al. 2012, Singer et al. 2017).

Autophototrophy acquisition through endosymbiont fixation and reduction involves numerous molecular and cellular reorganisations and modifications, and the steps leading to this process are still not fully understood (Keeling 2013). Nevertheless, some microbial eukaryotes have found ways to use photosynthesis for their own benefit without such profound alterations. In photosymbiosis, a heterotrophic eukaryote acquires a photosynthetic endosymbiont—either eukaryotic or bacterial—and takes advantage of the endosymbiont’s photosynthetic activity. This type of symbiosis is highly diverse in terms of host and endosymbiont biology, with examples found in most eukaryotic lineages, including ciliates, radiolarians (Rhizaria, TSAR), foraminiferans (Rhizaria, TSAR), centrohelids (Haptista), Katablepharida (Pancryptista), amoebozoans, and euglenids (see also Stoecker et al. 2009 and Yee et al. 2025 for a thorough review) and seem to be recurrent among species inhabiting oligotrophic environments where access to nutrients is usually limited (Leles et al. 2017). The most striking form of photosymbiosis is kleptoplasty (‘plastid stealing’), in which only the plastid of an ingested partner is retained and remains functional within the host cell (Cruz and Cartaxana 2022). This transient form of acquired phototrophy, where plastids are repeatedly sequestered from prey, offers a unique window into the intermediate stages of plastid establishment. Recent studies have documented especially compelling examples of kleptoplasty across dinoflagellates, euglenids, and centrohelids (Hehenberger et al. 2019, Karnkowska et al. 2023, Sørensen et al. 2023).

Collectively, these examples—from transient photosymbioses to fully established plastids—highlight the range of strategies by which eukaryotes have independently acquired photosynthetic capacity. While individual photosymbiotic events occur at the level of single species, in contrast to the broader diversification that followed the original plastid acquisitions, they underscore the pivotal role of endosymbiosis in enabling eukaryotic cells to acquire novel functions.

#### Nitrogen-fixing endosymbioses: expanding the functional capacity of eukaryotes

Nitrogen is a key limiting element for life. While it is essential in many biomolecules, such as nucleotides and amino acids, only some bacteria and archaea can fix atmospheric nitrogen into organic forms usable by other organisms (Boyd and Peters 2013), and most eukaryotes obtain organic nitrogen from external sources. However, well-documented examples exist in which N-fixation occurs directly inside a eukaryote, mediated by a prokaryotic endosymbiont. This includes multicellular organisms such as corals and plants, as well as single-celled eukaryotes, such as diatoms, radiolarians (Foster and Zher 2019), and dinoflagellates (Farnelid et al. 2010). A striking example is the haptophyte *Braarudosphaera bigelowii*, a unicellular marine alga with stable N_2_-fixing cyanobacteria *Ca*. Atelocyanobacterium thalassa (UCYN-A) as endosymbionts (Thompson et al. 2012). Study of this symbiotic relationship has revealed extraordinary biological integration, including synchrony of endosymbiont and host cell divisions and extensive import of nucleus-encoded proteins into the N_2_-fixing entity. These and other features have led to the recognition of this endosymbiont as a bona fide organelle, named the nitroplast, making *B. bigelowii* the first known eukaryote that fixes nitrogen (Coale et al. 2024). Taking into account the two photosynthetic organelles and the origin of mitochondria, this is the fourth independent event in which an endosymbiotic relationship with a prokaryotic partner has resulted in the acquisition of a new function in the eukaryotic cell (Massana 2024).

In addition to the nitroplast, there are other examples in which stable N_2_-fixing endosymbionts provide nitrogen to unicellular eukaryotes, especially among diatoms (Schvarcz et al. 2022). Moreover, these examples show significant differences, as compared with the integration process in *B. bigelowii*. For instance, N_2_-fixing endosymbionts in *Rhopalodia gibba* and certain *Epithemia* species are inherited vertically and provide nitrogen to the eukaryotic host through nitrogen fixation; however, none have been shown to import nucleus-encoded proteins (Prechtl et al. 2004, Frail et al. 2025), a hallmark often considered characteristic of organelles (Cavalier-Smith and Lee 1985, Theissen and Martin 2006). While the extent of nitrogen provisioning varies and is sometimes difficult to demonstrate, these associations highlight the role of N₂-fixing symbioses as a source of functional innovation in eukaryotes (Foster and Zher 2019).

### Protist thriving without oxygen: the role of endosymbionts

Adaptation to anaerobic conditions has occurred independently in most eukaryotic lineages throughout evolutionary history, involving various metabolic and mitochondrial modifications (Embley 2006, Muñoz-Gómez 2023). These transitions to anoxic and oxygen-depleted environments have been particularly frequent in ciliates, with numerous independent occurrences among both free-living and endobiotic gut species (Rotterová et al. 2022). Notably, most anaerobic ciliate species appear to harbour archaeal methanogens as endosymbionts (and presumably many also host bacterial sulphate reducers as ectosymbionts) (Fenchel and Finlay 1992), which has led to questions about the role of these endosymbionts in such adaptations. This is the case of the ciliate clade APM (Armophorea, Parablepharismea, and Muranotrichea) whose last common ancestor adapted to anaerobic conditions and diversified thereafter; and all of the studied species harbour endosymbionts (Rotterová et al. 2020). It has been proposed that the presence of these endosymbionts, which assimilate part of the byproducts of the host’s anaerobic metabolism, especially hydrogen, favoured longer survival in anoxia (Rotterová et al. 2020). The faster removal of this undesirable waste results in increased rates and efficiency of the host’s energetic metabolism (Fenchel and Finlay 1991). Thus, the presence of prokaryotic endosymbionts could have been essential to the loss of aerobic metabolic functions and the complete transition to obligately anaerobic metabolism (Rotterová et al. 2020).

Prokaryotic endosymbionts utilising hydrogen and other end-products are not exclusive to ciliates—they occur in various eukaryotic lineages where transitions to anoxic environments have taken place. For example, in the obligate anaerobic amoebozoan *P. schiedti*, which harbours two bacteria and one methanogenic archaeon as endosymbionts (Fig. 3d), methanogenesis with the consumption of hydrogen and formate (methanoate) has been shown to be essential for the survival of the amoebae; in the absence of methanogenic activity of the archaea, they die (Treitli et al. 2023). Interestingly, the related amoebozoan *Pelomyxa palustris* appears to rely on a similar dependency on its endosymbionts, although their taxonomic identity differs between the two cases (Gutiérrez et al. 2017). Other compelling examples of anaerobic protists harbouring methanogenic endosymbionts include a long list of lesser-known protists such as breviates (Obazoa, Amorphea) (Aguilera-Campos et al. 2025), heteroloboseans (Discoba) (Broers et al. 1993), as well as multiple lineages within Metamonada—oxymonads (Tokura et al. 2000), parabasalians such as the human parasite *Trichomonas* (Inoue et al. 2008), and Anaeramoebae (e.g. Táborský et al. 2017, Jerlström-Hultqvist et al. 2024). However, the energy demands of the endosymbionts and their metabolic integration remain largely unknown (Rotterová et al. 2020). Moreover, the diversity and metabolic capabilities of most endosymbionts of anaerobic microscopic eukaryotes have only recently begun to be understood. Unlike plastids and the nitroplast, the endosymbionts associated with the transition of eukaryotes to anaerobic environments appear to be highly diverse and to have arisen independently among and within the main groups of microbial eukaryotes. Nonetheless, specific closely related methanogenic taxa in ciliates seem more prone to form these associations (van Hoek et al. 2000, Schrecengost et al. 2024, Méndez-Sánchez et al. 2024). Expanding comparative investigations across independent transitions to anaerobic habitats will clarify the extent to which endosymbioses facilitated adaptation to oxygen-deficient conditions.

The most consequential respiratory endosymbiosis in eukaryotic evolution was the acquisition of mitochondria, which fundamentally reshaped cellular bioenergetics. Nevertheless, mitochondria are not the sole examples of respiratory endosymbionts in eukaryotes. Recent studies have identified *Ca*. Azoamicus ciliaticola, an endosymbiont of anaerobic ciliates that performs nitrate respiration via the denitrification pathway to generate ATP (Graf et al. 2021). This partnership represents an independent origin of respiratory endosymbiosis, distinct from mitochondria, with nitrate serving as the terminal electron acceptor. Initially, this symbiosis was reported in a single ciliate species from a lacustrine environment, suggesting a limited evolutionary impact. However, subsequent analyses have shown that, while apparently confined to a single clade within the Plagiopylea, this endosymbiosis is relatively widespread and has likely contributed to the ecological and evolutionary success of this lineage (Speth et al. 2024). Since the complete host range of these nitrate-respiring symbionts remains uncharacterised, it is plausible that their actual impact on eukaryotic evolution is broader than currently known.

#### Ecosystem-level consequences of protist endosymbioses

By modifying host cellular functions, endosymbioses expand metabolic capacities and allow eukaryotic hosts to exploit previously inaccessible ecological niches. These enhanced abilities not only facilitate host survival and diversification but also enable host–symbiont consortia to influence broader ecosystem processes. The huge impact of mitochondria and plastids on ecosystem functioning is indisputable. A wide range of other endosymbioses of unicellular organisms, such as those described above, most likely also have some ecosystem consequences (Fig. 3); however, the ecological significance of such partnerships remains largely unexplored (Beinart 2019).

Among endosymbioses with a clear influence on the carbon cycle, photosymbioses (including those involving cyanobacterial or algal partners) play a particularly important role, as—like permanent plastids—they supply organic carbon to their hosts (Decelle et al. 2015). Numerous protist lineages, predominantly in aquatic environments, engage in such symbioses, making it challenging to quantify their overall contribution to carbon cycling. Carbon fixation is typically measured at the single-cell level, complicating extrapolation to ecosystem or global scales. Further limitations arise from the difficulty of culturing many species and from the environment-dependent, often episodic nature of their photosynthetic activity. Consequently, generalisations about the contribution of most photosymbiotic systems to biogeochemical cycles remain limited.

The best-characterised examples of photosymbiosis occur in large host cells, such as foraminiferans and radiolarians, which can harbour numerous photosynthetic microalgae. These consortia can achieve primary productivity far exceeding that of an equivalent volume of free-living microalgae (Swanberg and Caron 1991), resulting in a substantial contribution of photosymbionts in radiolarians and foraminiferans to total primary production—ranging from approximately 1% of surface-water productivity to as much as 20% in certain regions (Caron et al. 1995). Overall, photosymbiosis, as a form of mixotrophy, is now broadly recognised as an important process shaping the functioning of aquatic environments and food webs (Caron 2016), although its full ecological significance still requires further investigation (Millette et al. 2023).

Just as photosymbioses influence the carbon cycle, nitrogen-fixing endosymbionts in microbial eukaryotes contribute to the nitrogen balance of the ecosystems they inhabit. Arguably, one of the most remarkable examples of nitrogen-fixing endosymbiosis is the nitroplasts of the haptophyte *Braarudosphaera* (Coale et al. 2024), discussed above, which have a substantial impact on oceanic nitrogen fixation. Initially described as the UCYN-A/haptophyte symbiosis, this association was thought to be largely restricted to tropical and subtropical oligotrophic waters. Coastal areas have generally been disregarded as important regions for N₂ fixation because they are often less nitrogen-limited than open-ocean oligotrophic regions (Holl and Montoya 2005). However, this symbiosis has been shown to contribute substantially to nitrogen fixation in temperate coastal waters (between 6% and up to 100%), with rates comparable to offshore regions (Turk-Kubo et al. 2021), showing that coastal ecosystems may be more relevant for diazotrophy than previously assumed. Moreover, the geographic range of this symbiosis has been reconsidered: CARD-FISH, which identifies specific microorganisms, combined with nanoSIMS, which tracks nutrient uptake at the single-cell level, revealed active nitrogen fixation far beyond previously recognised limits, including the Western Arctic, Bering, Chukchi, and Beaufort Seas (Harding et al. 2018). These findings confirm significant nitrogen assimilation in cold, high-latitude waters and considerably expand the known distribution of marine nitrogen fixers, challenging the long-held view that biological nitrogen fixation is confined to subtropical oceans. In the context of rapidly changing Arctic ecosystems, such high-latitude nitrogen-fixing symbioses may play an increasingly important ecological role.

As noted above, the nitroplast is the only known organelle derived from diazotrophic cyanobacteria related to *Crocosphaera*, in spite of these cyanobacteria also forming symbiotic associations with a variety of photosynthetic eukaryotes, including the diatoms *Epithemia* and *Climacodium* (Moulin et al. 2024). Such symbionts are commonly referred to as “diazoplasts”, although, unlike nitroplasts, they are not true organelles. The ecological significance of these symbioses was recently demonstrated in a tripartite association comprising the green macroalga *Cladophora glomerata*, epiphytic *Epithemia* diatoms, and their “diazoplasts” in a temperate river ecosystem (Marks et al. 2025). Using bulk isotope analysis, to measure overall isotope incorporation, nanoSIMS to track isotope uptake at the single-cell level, and density-gradient centrifugation for quantitative stable isotope probing to separate and quantify labelled cells, it was shown that a substantial fraction of diatom-fixed carbon is transferred to their nitrogen-fixing endosymbionts, fuelling most summertime nitrogen fixation in the river and accelerating energy flow through the food web (Fig. 3b). Although diazoplasts constitute only 3%–20% of the total nitrogen-fixing bacterial population in this ecosystem, their larger size relative to free-living diazotrophs results in their accounting for 74%–93% of the active nitrogen-fixer biovolume. Other cyanobacteria, such as *Richelia intracellularis*, also form symbiotic associations with diatoms, creating systems that exhibit high rates of N₂ and C fixation and are capable of forming blooms in nitrogen-limited, oligotrophic surface waters (Foster et al. 2022). Single-cell studies and host inhibition experiments indicate that hosts are the primary C fixers and likely regulate their symbionts’ N₂ fixation, while modelling suggests most fixed nitrogen is released in the upper water column, fuelling regional primary production. Collectively, these findings suggest that such endosymbioses can play a disproportionate role in driving biogeochemical cycles by supporting productive food webs in nitrogen-limited aquatic ecosystems (Foster et al. 2022, Schvarcz et al. 2022, Marks et al. 2025).

Cyanobacteria are not the only diazotrophs forming symbioses with protists. Recently, a non-cyanobacterial nitrogen-fixing prokaryote, *Ca*. Tectiglobus diatomicola—belonging to the Rhizobiales group of bacteria, better known for its role in rhizobia–legume symbioses on land—was described to associate with a diatom (Tschitschko et al. 2024). Remarkably, this endosymbiotic system was described in the tropical North Atlantic, a region responsible for roughly 20% of oceanic N₂ fixation. Measurements with nanoSIMS showed that 99% of the nitrogen fixed by the endosymbiont was transferred to the host. Notably, nitrogen-fixation rates for the *Ca*. T. diatomicola–diatom symbiotic system are comparable to the combined contribution of the most abundant cyanobacteria–diatom symbioses observed in the same waters, suggesting that non-cyanobacterial diazotrophs may account for a substantial part of the high nitrogen fixation in the tropical North Atlantic (Tschitschko et al. 2024). However, the role of this symbiosis likely extends beyond the tropical North Atlantic, as *Ca*. T. diatomicola is widespread and found across all major oligotrophic ocean regions. Its frequent association with larger size fractions further suggests symbioses with microbial eukaryotes. These observations indicate that symbiotic marine nitrogen-fixing Rhizobiales are significant contributors to oceanic N₂ fixation and play a crucial role in sustaining marine productivity and global CO₂ sequestration.

Diverse anaerobic protists form syntrophic associations with prokaryotic partners and together play a crucial role in the functioning of low-oxygen ecosystems. These ecosystems range from anoxic soils, waters, and sediments to intestinal tracts, such as cattle rumen. Depending on the symbionts, these syntrophic associations can be coupled to methanogenesis, sulphur reduction, or denitrification/nitrate reduction, thereby influencing methane emissions, driving reduced-sulphur cycling, and linking protist-mediated metabolism to the nitrogen cycle (Beinart et al. 2018a) (Fig. 3c). The impact on methane emissions is particularly profound and has been detected in shallow marine sediments (Fenchel and Finlay 2010, Beinart et al. 2018a), flooded soils (Schwarz and Frenzel 2005), and the rumen of cattle (López-García et al. 2022). The specific question, which is presently difficult to answer, is what proportion of ecosystem processes is carried out by host-associated versus free-living microbes. The ubiquity of protist-prokaryote associations in anoxic environments suggests that we may be overlooking a significant fraction of biogeochemical cycling by symbiotic prokaryotes. Importantly, the global significance of these associations is increasing as dissolved-oxygen concentrations decline in aquatic systems and anoxic zones expand in oceans and freshwater environments (Schmidtko et al. 2017, Zhang et al. 2025). Among environmentally relevant anaerobic endosymbioses, *Ca*. Azoamicus ciliaticola (Graf et al. 2021) provides a striking example, illustrating how much can be learned from a detailed study of protist—prokaryote endosymbioses (Fig. 3e). As previously mentioned, this endosymbiont functions as a respiratory symbiont and performs denitrification. Experimental studies have shown that denitrification rates in lake water are significantly elevated in the presence of the ciliate host (Graf et al. 2021), and when populations of symbiont-bearing ciliates are high—as observed in Lake Zug in Switzerland, with up to ∼24 500 cells per litre at 180 m depth—their cumulative contribution to nitrogen loss can be substantial. While this represents a single well-characterised example, environmental surveys have revealed many closely related endosymbionts in freshwater lakes, wastewater, and activated sludge systems worldwide, suggesting that this respiratory symbiosis may be more widespread and ecologically significant than previously recognised (Speth et al. 2024).

The diversity of protist endosymbionts observed across different hosts suggests effects that extend beyond individual organisms. By shaping nutrient cycling, microbial interactions, and energy flow, these partnerships can influence the structure and stability of microbial communities and, in turn, broader ecosystem processes. Understanding how these symbionts function in different hosts and environments will help reveal their broader ecological roles and their contribution to the resilience and functioning of ecosystems.

### Outlook

Research on interactions between protists and their prokaryotic and eukaryotic endosymbionts has advanced rapidly in recent years, driven by exciting discoveries and large-scale ecological studies. This progress has been enabled by extensive environmental sampling, improved culturing techniques, single-cell and -omics approaches, and advanced techniques such as nanoSIMS. Despite these advances, significant challenges remain. The most pressing questions are summarised below:

- **What are the origins, evolution and fate of protist endosymbionts?**

Understanding how endosymbiotic associations form, stabilise, or break down requires insights into the stages of symbiotic integration and the mechanisms that govern them. Current research approaches this from two directions: identifying naturally occurring intermediates that represent early or transitional states of integration (e.g. Karnkowska et al. 2023, Sørensen et al. 2023), and experimentally inducing novel endosymbioses to observe the processes of establishment (e.g. Giger et al. 2024).

- **What is the nature of protist endosymbiotic associations?**

Symbionts that provide well-defined benefits, such as carbon or nitrogen fixation, are relatively uncommon among protist endosymbionts. For most associations, the clear functional role of the symbiont(s) remains unidentified—if it exists at all. In many cases, the symbiont’s own benefits are equally uncertain. Determining these roles typically requires cultured host–symbiont systems that can be experimentally manipulated under controlled and varying environmental conditions (e.g. König et al. 2019, Iwai et al. 2019). The cultivation of protists, with or without their symbionts, was neglected for decades, but is now gaining momentum (del Campo et al. 2024), increasing the likelihood that additional symbiotic systems will soon become available in culture.

- **How widespread are endosymbioses among protist lineages?**

Systematic surveys are needed to map the diversity and prevalence of endosymbionts across protist lineages in natural environments. High-throughput methods, including single-cell genomics and environmental sequencing and sorting techniques, can enable analysis of many organisms directly from the environment. Such approaches will help reveal both known and previously undetected symbiotic associations across the tree of eukaryotes (e.g. Wittmers et al. 2025, Schulz et al. 2025).

- **What is the global impact of protist endosymbioses on ecosystem functioning?**

Understanding the ecological significance of endosymbioses requires direct measurement of symbiont activity compared to free-living counterparts. Cell-specific rates of carbon fixation, nutrient uptake, and other metabolic activities, combined with ecological analyses, will clarify how symbioses influence microbial activity and biogeochemical cycles. Integrating such measurements across environmental gradients will provide insights into the contribution of endosymbiotic protists to ecosystem functioning (e.g. Speth et al. 2024, Marks et al. 2025).
